# Adjustment disorder among undergraduate students at Prince Mohammad Bin Fahd University, Al Khobar, Saudi Arabia

**DOI:** 10.1192/j.eurpsy.2024.1697

**Published:** 2024-08-27

**Authors:** S. Aamir

**Affiliations:** Core Humanities and Social Sciences, Prince Mohammad Bin Fahd University, Al Khobar, Saudi Arabia

## Abstract

**Introduction:**

Adjustment disorder is characterized by an emotional or behavioral response to a stressful event or change in life. This condition can impact a student’s academic performance, social life, and overall well-being. Adjustment disorder with the stressor is a psychological response to identifiable stressors that result in the development of emotional or behavioral symptoms. These symptoms cause significant impairment in various areas of functioning, such as social, occupational, or academic performance.

Students with adjustment disorder may experience a range of symptoms, including feelings of sadness, anxiety, hopelessness, and a lack of concentration. They may also have trouble sleeping, feel overwhelmed, and struggle to cope with daily responsibilities. These symptoms can be triggered by various factors such as academic pressure, relationships, family issues, or cultural adjustments.

**Objectives:**

This study aims to determine the prevalence of adjustment disorder among undergraduate students and investigate the potential risk factors of stress that can lead to adjustment difficulties.

Recognize the signs of adjustment disorder, how to access support, and how to create a supportive environment, therefore, students can effectively manage this condition and thrive in their academic and personal lives.

To prioritize mental health and provide the necessary resources for students to navigate the challenges of adjustment disorder effectively.

**Methods:**

Adjustment Disorder‑New Model 20 (ADNM‑20) was used to assess prevalence of adjustment disorder among undergraduate students. It is a diagnostic tool used to assess adjustment disorder in individuals experiencing significant life stressors. The ADNM 20 is specifically designed to capture the nuanced manifestations of adjustment disorder with the stressor, enabling clinicians to make accurate assessments and develop targeted treatment plans.

**Results:**

Adjustment disorder is a real and impactful challenge and a common mental health condition among undergraduate students at Prince Mohammad Bin Fahd University, Al Khobar, Saudi Arabia.

**Conclusions:**

Adjustment disorder can significantly affect a student’s academic performance. The inability to focus, persistent feelings of distress, and a lack of motivation can lead to a decline in grades and overall achievement. By recognizing the signs, accessing support, and creating a supportive environment, students can effectively manage this condition and thrive in their academic and personal lives. It’s crucial to prioritize mental health and provide the necessary resources for students to navigate the challenges of adjustment disorder effectively.

**Disclosure of Interest:**

None Declared

